# Psychometric properties of the hematopoietic cell transplantation frailty scale: A prospective observational study

**DOI:** 10.1016/j.htct.2026.106471

**Published:** 2026-05-25

**Authors:** Luz Alejandra Lorca, Barbara Puga Larrain, Ivana Gonzalez Valdivia, Javiera Molina, Ivana Leao Ribeiro

**Affiliations:** a Hospital del Salvador, Servicio de Salud Metropolitano Oriente, Santiago de Chile, Chile; b Escuela de Kinesiología, Facultad de Ciencias de la Salud, Universidad Santo Tomás, Chile; c Departamento de Kinesiología, Facultad de Ciencias de la Salud, Universidad Católica del Maule, Talca, Chile

**Keywords:** Frailty, Hematopoietic stem cell transplantation, Psychometrics, Frailty scale, Hematologic neoplasms

## Abstract

**Introduction:**

Frailty is common in patients undergoing hematopoietic stem cell transplantation, thereby increasing toxicity risk and reducing post-transplant survival. This study evaluated the psychometric properties of the Hematopoietic Cell Transplantation Frailty Scale to assess transplant candidates. Additionally, it characterized the participants` sociodemographic and clinical profiles.

**Methods:**

One hundred and two patients with hematologic malignancies participated in the study. Reliability was evaluated through internal consistency (Cronbach’s alpha), factorial analysis with varimax rotation, and construct validity via hypothesis testing. Scale items were grouped in three dimensions: (1) objective, (2) subjective, and (3) laboratory parameter assessments.

**Results:**

Participants had a mean age of 40.5 ± 14.5 years; 15 (14.7%) were classified as ‘frail,’ 48 (47.1%) as ‘pre-frail,’ and 39 (38.2%) as ‘fit.’ Multiple myeloma was the most common diagnosis (32.4%). Internal consistency was acceptable (α = 0.63). The first dimension explained 55.3% of the variance, while the second contributed 31.5%, totaling 86.8% of the explained variance. Construct validity confirmed only one hypothesis: patients with a performance status below 90 had significantly higher frailty scores than those with a performance status of 90–100 (p-value <0.01). Other hypotheses comparing frailty classification (frail, pre-frail, and fit) across Disease Risk Index categories (low-moderate, high-very high, not evaluable), Hematopoietic Cell Transplantation Comorbidity Index (<3, ≥3), transplant type (autologous, allogeneic), and age groups (18–59, ≥ 60 years) were rejected (p-value >0.05).

**Conclusion:**

The Chilean version of the Hematopoietic Cell Transplantation Frailty Scale demonstrates adequate reliability and validity, supported by a strong factorial structure to assess transplant candidates. Frailty was associated with a lower performance status, but not with the Disease Risk Index, the Comorbidity Index, transplant type or age.

## Introduction

Hematopoietic stem cell transplantation (HSCT) is used with curative intentions for hematologic neoplasms and other potentially lethal diseases [1]. Improvements in clinical practice, along with the increase in the population life expectancy, have expanded the indications of both autologous and allogeneic HSCT [2].

Frailty is a syndrome characterized by reduced physiological reserve in patients, which in HSCT patients increases the risk of toxicities and reduces post-transplant survival [3, 4, 5, 6]. Additionally, frailty has been associated with poor post-HSCT outcomes in both young and old individuals [7].

Thus, identifying and treating frailty in HSCT candidates can improve final transplant outcomes. Different tools have been developed to diagnose frailty, most of which are adapted from validated geriatric frailty scales [6,8]. Nevertheless, a significant number of HSCT candidates are not geriatric patients, limiting the applicability of these scales for younger individuals [6,8].

While frailty scales are being developed for implementation in the HSCT setting, there is still no consensus on the best assessment methodology, target population, cost-effectiveness, and predictive accuracy [9].

Professionals at Princess Margaret's Hospital developed the Hematopoietic Cell Transplantation Frailty Scale, a tool to assess frailty and functionality in HSCT candidates regardless of age. It is a multidimensional assessment composed of eight items specifically selected to be administered in the daily clinical practice, with no additional waiting time for patients and using existing human resources [9,10].

The HCT Frailty Scale corresponds to a quick and easy-to-implement tool that combines functional variables related to frailty along with laboratory biomarkers that categorize adult HSCT candidates into three categories: ‘fit,’ ‘pre-frail’ and ‘frail’ [10].

The HCT Frailty Scale still lacks psychometric studies in the Chilean population. As the validation process is dynamic and continuous, frailty scales acquire greater relevance as they advance and deepen with psychometric studies that allow the strengthening of their use in the daily clinical practice [11]. Therefore, the use of validated instruments tailored to the specific population is recommended for clinical applications. Thus, the objective of this study is to evaluate the psychometric properties such as reliability and validity of the HCT Frailty Scale in a sample of Chilean HSCT candidates, and, subsequently, characterize their sociodemographic and clinical profiles.

## Methods

This observational study with a cross-sectional design followed the Consensus-based Standards for the selection of health Measurement Instruments (COSMIN) guidelines [12].

### Participants

This psychometric study included 102 patients (aged ≥18 years) diagnosed with onco-hematological diseases who were candidates for autologous or allogeneic HSCT at a public hospital in Santiago, Chile.

The exclusion criteria were people with disabilities and cognitive deficits that limited their ability to understand instructions and sign an informed consent. Additionally, patients with insufficient comprehension of Spanish were excluded. The Mini-Mental Abbreviated test was used to evaluate the cognitive dimension [13].

### Procedure

Patients were recruited during their first consultation with a hematologist in the HSCT program. Those who met the eligibility criteria were invited to participate in this study. The study objectives and the details of their participation were explained to those who voluntarily agreed to participate and signed an informed consent form.

The evaluations, conducted by two physical therapists with clinical experience of HSCT patients, were performed in a physical medicine and rehabilitation polyclinic between December 2023 and January 2025.

A convenience sampling method was used. The sample size was estimated based on the main objective of this study, which was to evaluate reliability in psychometric studies. It was calculated that a minimum of 100 participants was required to obtain reliable estimates of Cronbach's alpha [14].

This study adhered to ethical standards of the Declaration of Helsinki and was approved by the scientific ethics committee of the Eastern Metropolitan Health Service (December 5, 2023). The HCT Frailty Scale was administered approximately 45 days before transplantation as part of the routine pre-transplant evaluation. Patients identified as ‘pre-frail’ or ‘frail’ were referred to a structured prehabilitation program, in line with institutional clinical care protocols.

### Hematopoietic cell transplantation frailty scale

This scale was created by professionals at the Princess Margaret Cancer Center, Toronto, Canada, and is composed of eight items: Clinical Frailty Score (CFS), Instrumental Activities of Daily Living (IADL) test, Hand Grip Score (GS), Timed Up and Walk Test (TUGT), Self-Reported Health Question (SRH-Q), Single Falls Question, Self-Reported Health Levels (SRH-Q), and the measurement of the serum albumin and C-reactive protein (CRP) levels [9,10].

A Jamar® hydraulic dynamometer (J A Preston Corporation, New York, USA) was used for the handgrip strength test.

The HCT Frailty Scale allows patients who are candidates for HSCT to be categorized as ‘fit’, ‘pre-frail’ and ‘frail’ according to the score resulting from the sum of the weighted values of the eight items of the scale.

### Karnofsky functional status scale (KPS)

A numerical scale was used to quantify the functional status of patients concerning their degree of independence in daily activities and self-care needs. Patients are assessed using an 11-category scale ranging from 0 to 100, where a higher score indicates greater ability to perform daily activities [15]. In this study, patients were grouped according to the following ranges: <90 and 90–100.

### Disease risk index (DRI)

The Disease Risk Index (DRI) is a validated tool to categorize HSCT patients with hematologic malignancies. Its purpose is to stratify patients into broad categories of disease risk. The following categories were used in this study: low-moderate, high-very high, and not assessable [16].

### Hematopoietic cell transplantation-comorbidity index (HSCT-CI)

Hematopoietic Cell Transplantation Comorbidity Index (HSCT-CI), also known as the Sorror Comorbidity Index, is a score that estimates the risk of transplant mortality based on patient comorbidities and the type of conditioning indicated. Based on the score obtained, patients receive scores of 0 to 4. The following ranges were used in this study: <3 and ≥3 [17].

### Sociodemographic and clinical background

Information was compiled on variables (age, sex, schooling, marital status, employment status, occupation) and clinical variables (treatments received, smoking, alcohol consumption, weight, height, body mass index [BMI], diagnosis, transplant type, treatments received).

### Statistical analysis

The data were tabulated in an Excel spreadsheet and analyzed with the Statistical Package for Social Sciences (SPSS) version 30 software. Descriptive statistics were reported as frequency, mean, median, and interquartile range.

Regarding psychometric properties, reliability was assessed through internal consistency and construct validity using hypothesis testing.

Cronbach's alpha analysis was used to evaluate the internal consistency and dimensionality of the scale employing the values described by Loewenthal [18]. These authors suggested that an internal consistency of 0.60 was acceptable for scales with less than ten items such as the HCT Frailty Scale (eight items). In addition, a factor analysis was conducted to identify the factor loading and instrument dimensions, considering Barlett's test assumptions (p-value <0.05), and using the Kaiser-Meyer-Olkin test.

For these analyses, the eight items of the scale were grouped into three dimensions: (1) subjective dimension, grouping items 2, 5, and 6, (2) objective dimension, grouping items 1, 3, and 4, and (3) laboratory parameters grouping items 7 and 8.

Hypothesis testing using the Mann-Whitney U test was used to evaluate the construct validity of the HCT Frailty Scale. Five hypotheses were tested employing this test:

(1) Patients with a Karnofsky Performance Status (KPS) <90 have higher frailty scores compared to those with a performance status of 90 to 100.
(2) Patients with high to very high DRI have higher frailty scores compared to those with a low to moderate DRI.
(3) Patients with HSCT-CI ≥3 have a higher frailty score compared to those with HSCT-CI <3.
(4) Patients who are candidates for allogeneic HSCT have higher frailty scores compared to those who are candidates for autologous HSCT.
(5) Older adult patients (≥60 years) have higher frailty scores compared to younger adult patients (18–59 years).

## Results

### Sociodemographic and clinical characterization of the participants

A total of 102 patients participated, with an average age of 40.5 years. Fifty-two (51.0%) were women and fifty (49.0%) were men. The most prevalent diagnosis was multiple myeloma in 33 patients (32.4%), followed by acute lymphoblastic leukemia in 22 patients (21.6%). Of the total sample, fifty-nine patients (57.8%) were candidates for autologous HSCT, 34 (33.3%) for allogeneic haploidentical HSCT, and nine (8.8%) for allogeneic HSCT with an identical family donor (IFD). Regarding frailty, 15 (14.7%) individuals were categorized as ‘frail’, 48 (47.1%) as ‘pre-frail’ and 39 (38.2%) as ‘fit’. Table 1 presents all the sociodemographic and clinical characteristics of the participants (Table 1).

**Table 1:** Characterization of study participants (n = 102).

Variable	
**Sex** – n (%)	
Female	52 (51.0)
Male	50 (49.0)
**Age - years** – median ± SD (range)	40.5 ± 14.5 (37.6–43.4)
18–59 years – n (%)	98 (96.1)
≥ 60 years – n (%)	4 (3.9)
**Anthropometric Measurements** – median ± SD (range)	
Height - m	1.64 ± 0.10 (1.62–1.66)
Weight - kg	74.8 ± 17.5 (71.4–78.3)
Body Mass Index - kg/cm^2^	27.4 ± 5.2 (26.4–28.4)
**Diagnosis** – n (%)	
Plasma cell disorders: (Multiple Myeloma; Amyloidosis)	34 (33.3)
Acute Leukemias: (Acute Lymphoblastic Leukemia; Acute Myeloid Leukemia; Acute Leukemia of Ambiguous Lineage)	34 (33.3)
Mature Lymphoproliferative disorders (Hodgkin Lymphoma; Non-Hodgkin Lymphoma)	25 (24.5)
Myeloproliferative neoplasms (Chronic Myeloid Leukemia)	2 (2)
Myelodysplastic Neoplasms/Aplastic anemia (Aplastic anemia, MDS, Hypoplastic MDS)	7 (6.9)
**Type of Treatment** – n (%)	
Chemotherapy/Radiotherapy/Immunotherapy	2 (2.0)
Chemotherapy/Immunotherapy	7 (6.9)
Chemotherapy/Radiotherapy	8 (7.8)
Chemotherapy	78 (76.5)
Immunotherapy	5 (4.9)
Not Declared	1 (1.0)
**Type of HSCT** – n (%)	
Autologous	59 (57.8)
Allogeneic - Identical Family Donor	9 (8.8)
Allogeneic - Haploidentical	34 (33.4)
**DRI** – n (%)	
Low-Moderate	78 (76.5)
High-Very High	19 (18.5)
Not Evaluable	5 (4.9)
**Karnofsky Score** – n (%)	
90–100	52 (51.0)
<90	50 (49.0)
**HSCT-CI** – n (%)	
< 3	91 (89.2)
> = 3	11(10.8)
**Patient Categorization According to HCT Frailty Scale** – n (%)	
Frail	15 (14.7)
Pre-frail	48 (47.1)
Fit	39 (38.2)

Mean ± Standard Deviation [Lower Limit; Upper Limit, 95% Confidence Interval].

DRI: Disease Risk Index; HSCT-CI: Hematopoietic Cell Transplantation–Specific Comorbidity Index.

### Psychometric properties

#### Internal consistency and dimensionality

A Cronbach's α of 0.63 was obtained for the total scale, indicating acceptable internal consistency. For the objective dimension which grouped items 1, 3 and 4, Cronbach's α was 0.61. For the subjective dimension which grouped items 2, 5, and 6, Cronbach's α was 0.40 and for laboratory parameters that grouped items 7 and 8, the Cronbach's α was 0.10 (Table 2).

**Table 2:** Reliability of the HCT Frailty Scale (n = 102).

HCT Frailty Scale Dimensions	Item	Cronbach's Alpha
Objective Assessment	Item 1: Clinical Frailty Scale Item 3: Timed Up and Go Test (TUGT) Item 4: Handgrip Strength	0.61
Subjective Assessment	Item 2: Instrumental Activities of Daily Living (IADL) Item 5: Self-reported Health Question Item 6: Falls in the Last Six Months	0.40
Laboratory Parameter Assessment	Item 7: Serum Albumin Level Item 8: C-Reactive Protein	0.10

Total Cronbach's Alpha of the HCT Frailty Scale: 0.63.

#### Factor analysis and varimax rotation

**Figure 1:**
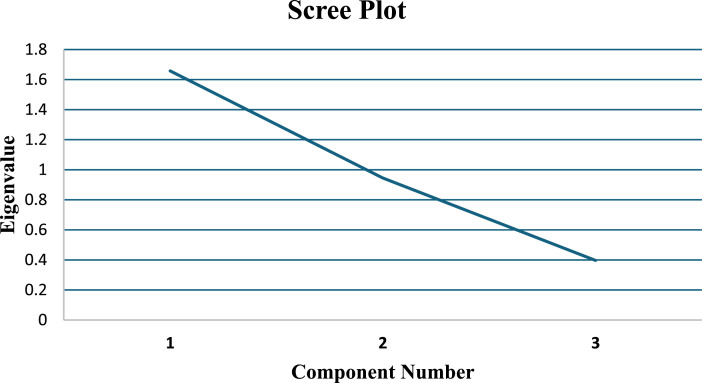
Scree plot of the scale dimensions.

Regarding the analysis of the scale components with varimax rotation, the Kaiser-Meyer-Olkin test of sample adequacy produced a score of 0.74, indicating acceptable adequacy. Bartlett’s test of sphericity was significant (p-value <0.01), supporting the relevance of performing the factor analysis. The first dimension (objective evaluation) accounted for 55.3% of the variance, making it the strongest factor in the scale. The second dimension (subjective evaluation) accounted for 31.5%, bringing the cumulative variance explained by both dimensions to 86.7%. Finally, the third dimension had a low eigenvalue (0.39) and explained only 13.2%, suggesting that it is a weak factor, that is, its component items contribute less to the overall scale (Table 3). Figure 1 represents the sedimentation graph, which shows that the slope decreases from factor 2 of the scale, specifically in factor 3, which presented a low eigenvalue (Figure 1 & Table 3).

**Table 3:** Variance explained by extracted factors.

HCT Frailty Scale Dimensions	Eigenvalue	% of Variance	Cumulative %
Objective Assessment Item 1: Clinical Frailty Scale Item 3: Timed Up and Go Test (TUGT) Item 4: Handgrip Strength	1.65	55.26	55.26
Subjective Assessment Item 2: Instrumental Activities of Daily Living (IADL) Item 5: Self-reported Health Question Item 6: Falls in the Last Six Months	0.94	31.51	86.77
Laboratory Parameter Assessment Item 7: Serum Albumin Level Item 8: C-Reactive Protein	0.39	13.22	100.00

Extraction Method: Principal Component Analysis. Rotation Method: Varimax Normalization with Kaiser-Meyer-Olkin test = 0.74, Bartlett’s Sphericity Test p-value < 0.01.

1. Objective assessment: Item 1: Clinical Frailty Scale; Item 3: Timed Up and Go Test (TUGT); Item 4: Handgrip strength. 2. Subjective assessment: Item 2: Instrumental Activities of Daily Living (IADL); Item 5: Self-reported health question; Item 6: Falls in the last six months. 3. Laboratory parameter assessment: Item 7: Serum albumin level; Item 8: C-reactive protein.

#### Construct validity

Construct validity confirmed only one hypothesis: patients with a Karnofsky performance status <90 had higher frailty scores compared to those patients with performance status of 90 to 100 (u = 2048.0; p-value <0.01).

The remaining four hypotheses were rejected (p-value >0.05). There was no statistically significant difference for the subgroups: DRI (K = 3.87; p-value = 0.14), HSCT-CI (U = 349.000; p-value = 0.07), type of HSCT (U = 1.252; p-value = 0.90), and age (K = 5.373; p-value = 0.06) (Table 4).

**Table 4:** Prevalence of the Hematopoietic Cell Transplantation Frailty Scale by Disease Risk Index, performance status, Hematopoietic Cell Transplantation Comorbidity Index, and transplantation type.

	Frail	Pre-frail	Fit	Test value; P-value

**Hematopoietic Cell Transplantation (HSCT)**	Autologous:	Autologous:	Autologous:	U = 1.252;
	6 (40)	32 (66,.7)	21 (53.8)	p-value = 0.90
	Allogeneic:	Allogeneic:	Allogeneic:	
	9 (60)	16 (33.3)	18 (46.2)	
**Performance status (Karnofsky Score)**	90–100: 14 (13.7)	90–100: 28 (27.5)	90–100: 10 (9.8)	U = 2048.0;
	<90: 1 (1.0)	<90: 20 (19.6)	<90: 29 (28.4)	p-value <0.01
**Disease Risk Index (DRI)**	Low-Moderate:	Low-Moderate:	Low-Moderate:	K = 3.87;
	12 (11.8)	37 (36.3)	29 (28.4)	p-value = 0.14
	High-Very high:	High-Very high:	High-Very high:	
	3 (2.9)	10 (9.8)	6 (5.9)	
	Not Evaluable:	Not Evaluable:	Not Evaluable:	
	0 (0)	1 (1.0)	4 (3.9)	
**Hematopoietic Cell Transplantation–Specific Comorbidity Index (HSCT-CI)**	<3	<3	<3	U = 349.000;
	13 (86.7)	40 (83.3)	38 (97.4)	p-value = 0.07
	> = 3	> = 3	> = 3	
	2 (13.3)	8 (16.7)	1 (2.6)	
**Age**	18–59 years	18–59 years	18–59 years	K = 5.373;
	≥ 60 years	≥ 60 years	≥ 60 years	p-value = 0.06

The data are expressed as frequency (percentage). U: Mann-Whitney test; K: Kruskal-Wallis test.

HSCT: Hematopoietic Stem Cell Transplantation; DRI: Disease Risk Index; HSCT-CI: Hematopoietic Cell Transplantation Comorbidity Index.

**Figure 2:**
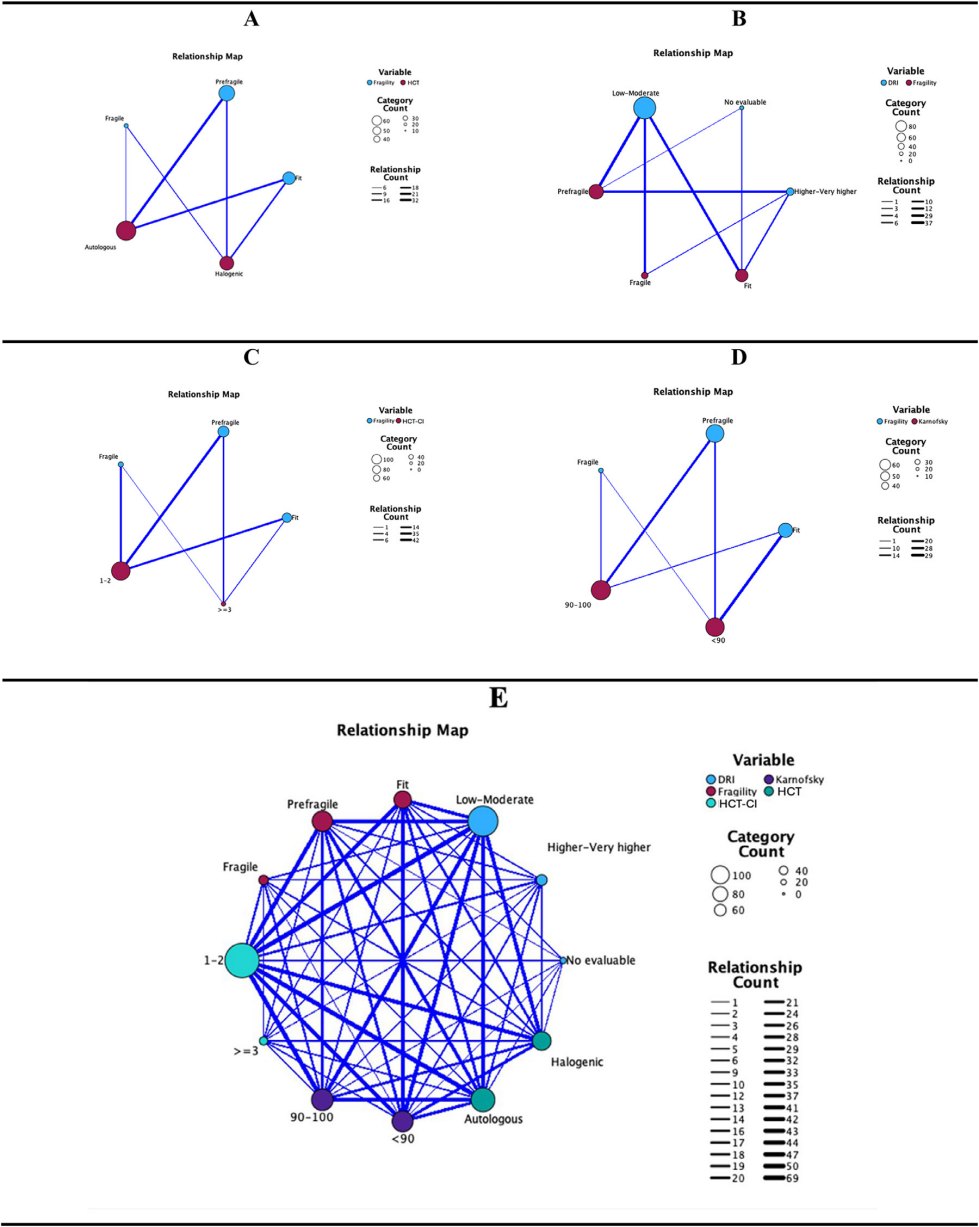
Relationship map between frailty categories according to the HCT Frailty Scale and DRI, type of HSCT, Karnofsky score, HSCT-CI, and all variables combined.

Complementary to the hypothesis tests, Figure 2 represents the relationship map between frailty categories and other variables (DRI, type of HSCT, Karnofsky score, and HSCT-CI). In general, the connections established in the map reflect the strength of the association between the variables; the thickness of the lines indicates the magnitude of these associations. Although the aforementioned hypotheses were rejected, autologous HSCT candidates tend to be pre-frail compared to allogeneic candidates (Figure 2A). Low DRI is associated with all three categories of frailty concerning the other indices in this category (Figure 2B), as is a HSCT-CI index between 1–2 (Figure 2C). Additionally, the lower performance status score is more strongly associated with pre-frail and fit individuals (Figure 2D). Regarding patient frailty categorization, Supplemental Table 1 presents the classification of each domain of the HCT Frailty Scale as normal or abnormal, according to established cutoffs. These individual scores were summed to obtain a total frailty score (range: 0–10.5), which allows categorization of patients as ‘fit’, ‘pre-frail’, or ‘frail’.

A: Relationship between frailty and HSCT. B: Relationship between frailty and DRI. C: Relationship between frailty and HSCT-CI. D: Relationship between frailty and performance status. E: Relationship between frailty and HSCT, DRI, HSCT-CI, and performance status

## Discussion

This study demonstrates that the Chilean Spanish version of the HCT Frailty Scale is adequate in terms of internal consistency and construct validity in the assessment of frailty in HSCT.

The sociodemographic background of this study identified a young population with a mean age of 40.5 ± 14.5 years, exhibiting some degree of frailty. This finding is consistent with previous studies that indicated that frailty is not exclusive to the geriatric population [3,5,10]. Additionally, patients with hematological malignancies who are candidates for HSCT may also exhibit this syndrome [5,9,10].

In terms of frailty, the majority of participants were categorized as ‘prefrail’ (n = 48; 47.1%) followed by ‘fit’ (n = 39; 38.2%) and a minority as ‘frail’ (n = 15; 14.7%). Similar results were reported in studies conducted by the original authors of the HCT Frailty Scale. In their initial consultation assessment, 53.4% were classified as ‘pre-frail’, 28.8% as ‘fit’ and 18.2% as ‘frail’ [5,10,19].

The criteria used to assign patients to these categories are summarized in Supplementary Table 1, which details the classification of each HCT Frailty Scale domain (normal versus abnormal) based on established cutoffs, and how these scores determine the overall frailty status.

Regarding the psychometric properties, the scale demonstrated acceptable internal consistency, with Cronbach's alpha values of 0.63 for the total scale. Internal consistency is a measure of the correlation between items in a measurement instrument. Generally, values are acceptable between ≥0.70 and ≤0.95. However, when interpreting the value of the coefficient of internal consistency, it is convenient to take into account the length of the scale. It is generally recommended that scales with ten homogeneous items should achieve an adequate reliability [20]. Nevertheless, many modern scales comprise fewer than ten items. In these cases, internal consistency can still be acceptable with values below 0.70. This was the case in this study, where the HCT Frailty Scale (with eight items) achieved a Cronbach's alpha of 0.63, which is considered acceptable [18].

In this study, construct validity confirmed only one hypothesis related to performance status with results similar to those reported in the study by Salas et al., who found that patients with a KPS <90 were more likely to be frail than those with a higher KPS [9].

Contrary to expectations, the results of this study indicate that frailty is a common syndrome in both patients with HSCT-CI ≥ 3 and those with a HSCT-CI <3. This finding is consistent with Salas et al., who reported that frailty was independent of the initial comorbidity index [19]. This was also the case for patients with both high-very high and low-moderate DRI.

Regarding age, the results of this study show that comparable levels of frailty can be found in both younger (under 60 years) and older adults (≥60 years). Rejecting the initial hypothesis, it was found that young adult candidates for HSCT may also be frail due to multiple factors, such as underlying disease, prior treatments, and existing complications [10,21,22].

Finally, the type of HSCT (autologous or allogeneic) did not influence the frailty score. Similar results were obtained in the study by Singh et al., where similar distributions of frailty domains were observed for autologous and allogeneic HSCT [23]. Considering that most studies on frailty in HSCT have focused exclusively on allogeneic HSCT [10,24] the findings of the present study are noteworthy as a significant percentage of autologous HSCT candidates (57.8%), who may also present pretransplant frailty, was included.

One of the limitations of this study was the absence of published psychometric studies of the HCT Frailty Scale, which restricted the possibility of comparing these findings with other studies. However, a nationally representative sample was involved, evaluating HSCT candidates from all regions of the country.

In addition, it is important to note that although frailty assessments are currently recommended prior to HSCT [4,24,25], they have not yet been systematically incorporated into pre-HSCT evaluations.

Likewise, the implementation of pre-HSCT frailty assessments remains an area of emerging interest and active research [1]. Therefore, this study represents a breakthrough in the area, demonstrating how frailty interacts with key factors in HSCT candidates, and identifying clinically relevant patterns for decision-making in this population.

Finally, as frailty status is dynamic in nature, i.e., the transition from ‘fit’ to ‘pre-frail’ and ‘frail’ is not always linear, it is potentially reversible and can be modified through targeted interventions [26]. Therefore, routine frailty assessment and monitoring is gaining importance in HSCT patients [1,18] and could be a preliminary step in designing specific, multidisciplinary interventions, including prehabilitation and post-HSCT rehabilitation programs. Therefore, the management of this syndrome should focus on its identification, treatment, and prevention of its potential consequences [27] aiming to reduce the negative impact of HSCT on the patient’s quality of life, morbidity, and mortality.

## Conclusions

The Chilean version of the HCT Frailty Scale has demonstrated adequate reliability and validity, with a factorial structure that supports its use in assessing frailty in HSCT candidates. Frailty was associated with lower performance status, but not with DRI, HSCT-CI, transplant type, or age.

## Data availability

The data that support the findings of this study are available from the corresponding author upon reasonable request.

## Funding

None.

## Conflicts of interest

The authors declare no conflicts of interest.

## Appendix Supplementary materials

Image, application 1
