# Supplementary material for: Psychometric properties of the hematopoietic cell transplantation frailty scale: A prospective observational study
Source: Hematol Transfus Cell Ther. 2026 May 25;48(3):106471. doi: 10.1016/j.htct.2026.106471 (PMC13223521; doi:10.1016/j.htct.2026.106471)
Supplement: Supplementary file 1 [file mmc1.docx]

**Supplementary Table 1:** Hemopoietic Cell Transplantation Frailty Scale: Domain Classification (Normal versus Abnormal)

| **Item** | **Abnormal** | **Normal** |
| --- | --- | --- |
| Clinical Frailty Score (CFS) | 1.5 points ≥ 3 (frail) | 0 points 1–2 (not frail) |
| Instrumental Activities of Daily Living (IADL) | 1 point ≥ 1 limitation | 0 points No limitation |
| Timed Up and Go Test (TUGT) | 1.5 points >10 seconds | 0 points ≤10 seconds |
| Hand Grip Strength (HGS) | 1 point <16 kg (women) <26 kg (men) | 0 points |
| Self-Rated Health (SRH) | 1 point Fair or poor | 0 points Excellent, very good, or good |
| Falls in the last 6 months | 1 point Yes | 0 points No |
| Serum Albumin Level | 1.5 points <38 g/L | 0 points |
| C-Reactive Protein (CRP) | 2.0 points ≥11 mg/L | 0 points |

Total Score: 0-10.5

Frailty categorization: Fit: ≤1; Pre-frail: 1.5-5.0; Frail: ≥5.5
